# Supplementary material for: Modelling Spatiotemporal Patterns of Lyme Disease Emergence in Québec
Source: Int J Environ Res Public Health. 2021 Sep 14;18(18):9669. doi: 10.3390/ijerph18189669 (PMC8470240; doi:10.3390/ijerph18189669)
Supplement: Supplementary file 1 [file ijerph-18-09669-s001.zip › ijerph-1338873-supplementary final.pdf]

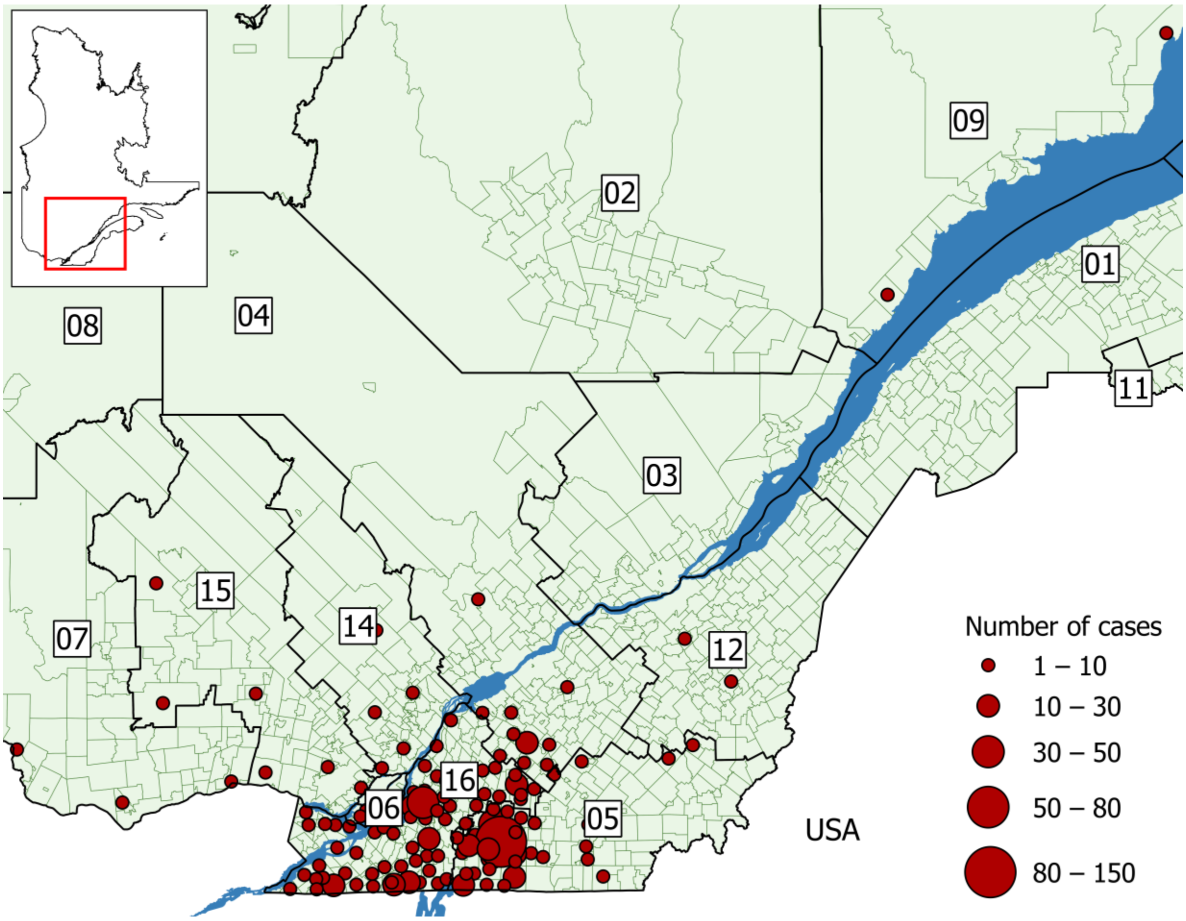

Figure S1. Cumulative number of Lyme disease cases in Québec acquired locally, 2006–2018 by date of symptom onset (n = 693). Sociosanitary regions: 01) Bas-Saint-Laurent; 02) Saguenay – Lac-Saint-Jean; 03) Capitale-Nationale; 04) Mauricie et Centre-du-Québec; 05) Estrie; 06) Montréal; 07) Outaouais; 08) Abitibi-Témiscamingue; 09) Côte-Nord; 11) Gaspésie – Îles-de-la-Madeleine; 12) Chaudière-Appalaches; 14) Lanaudière; 15) Laurentides; 16) Montérégie.

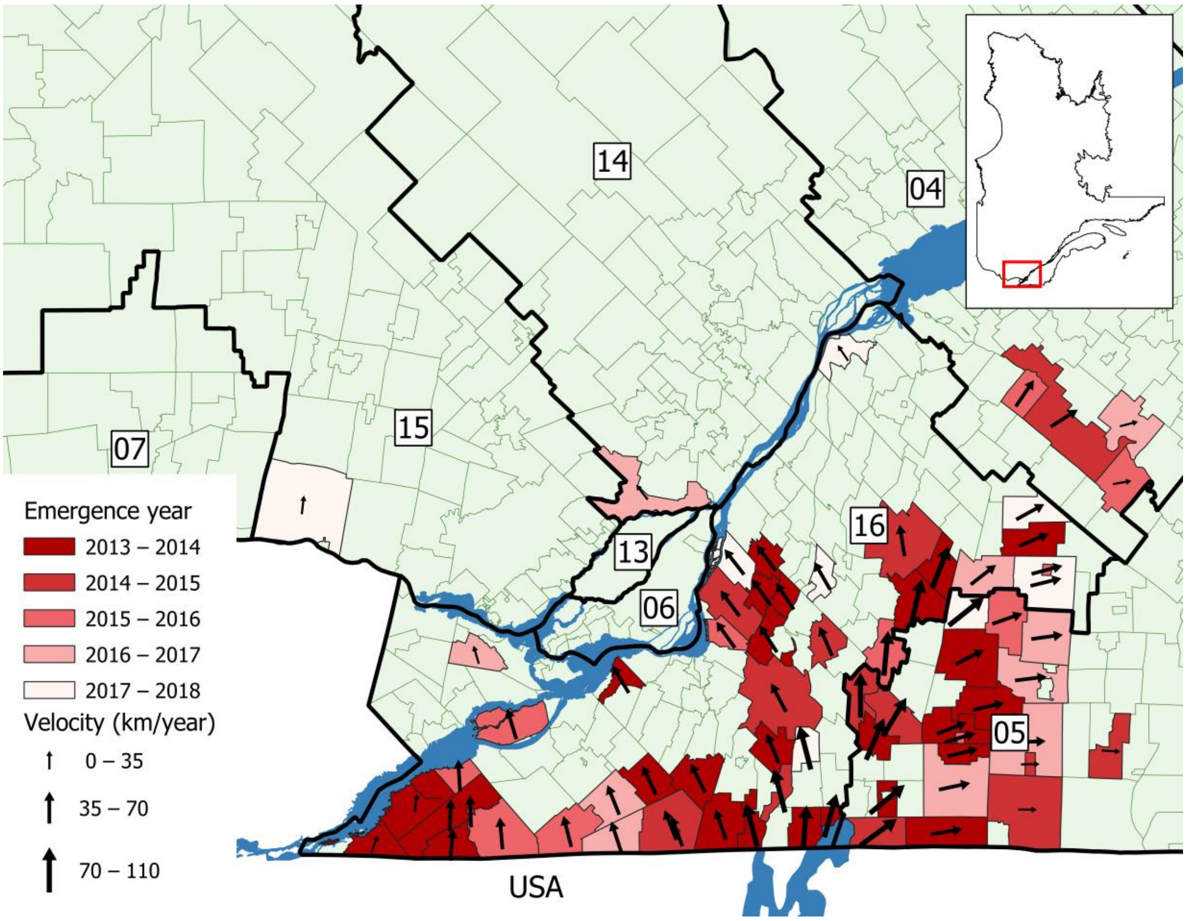

Figure S2. Lyme disease emergence velocity in Québec in municipalities having declared at least two cases in the last five years (n = 70) by date of symptom onset (2013–2018). Sociosanitary regions: 04) Mauricie et Centre-du-Québec; 05) Estrie; 06) Montréal; 07) Outaouais; 13) Laval; 14) Lanaudière; 15) Laurentides; 16) Montérégie.

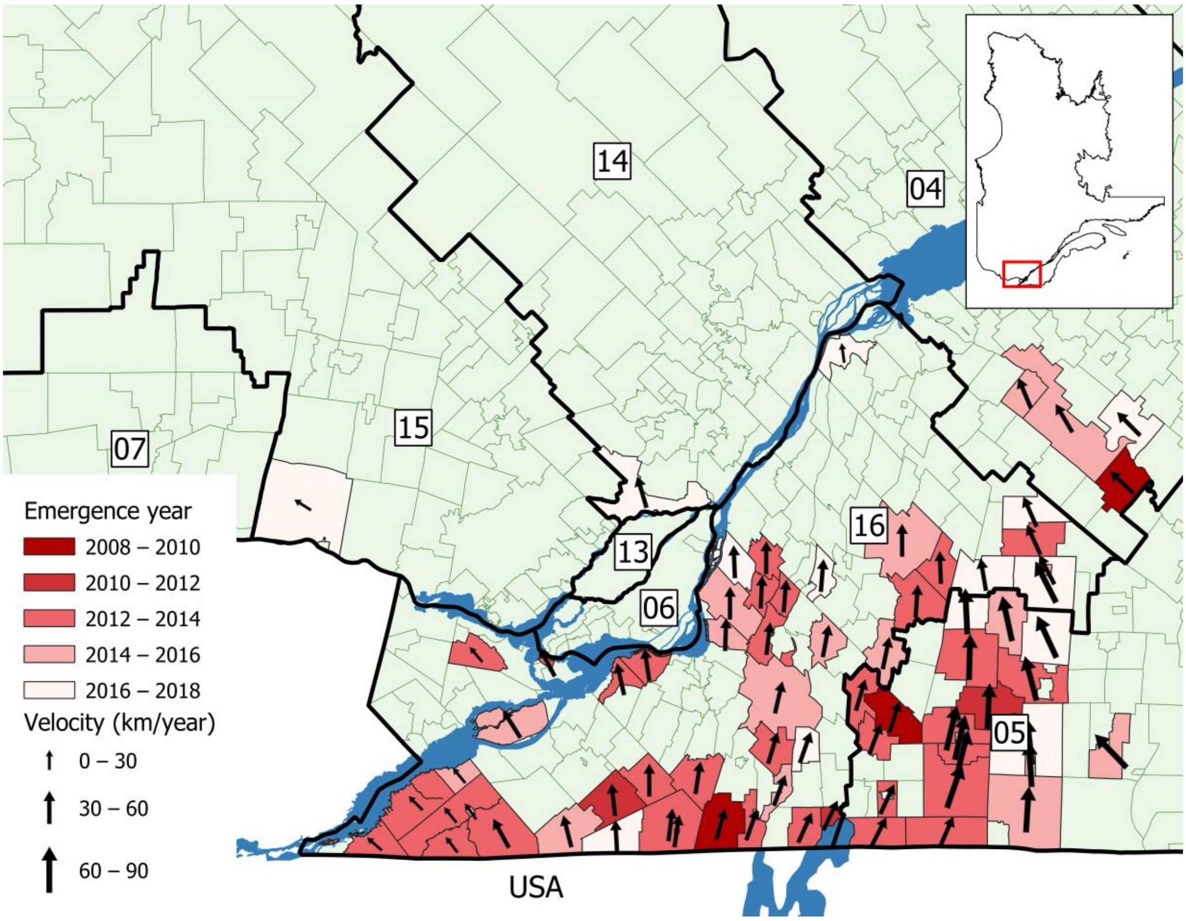

Figure S3. Lyme disease emergence velocity in Québec in municipalities having declared at least two cases (n = 72) by date of symptom onset (2006–2018). Sociosanitary regions: 04) Mauricie et Centre-du-Québec; 05) Estrie; 06) Montréal; 07) Outaouais; 13) Laval; 14) Lanaudière; 15) Laurentides; 16) Montérégie.

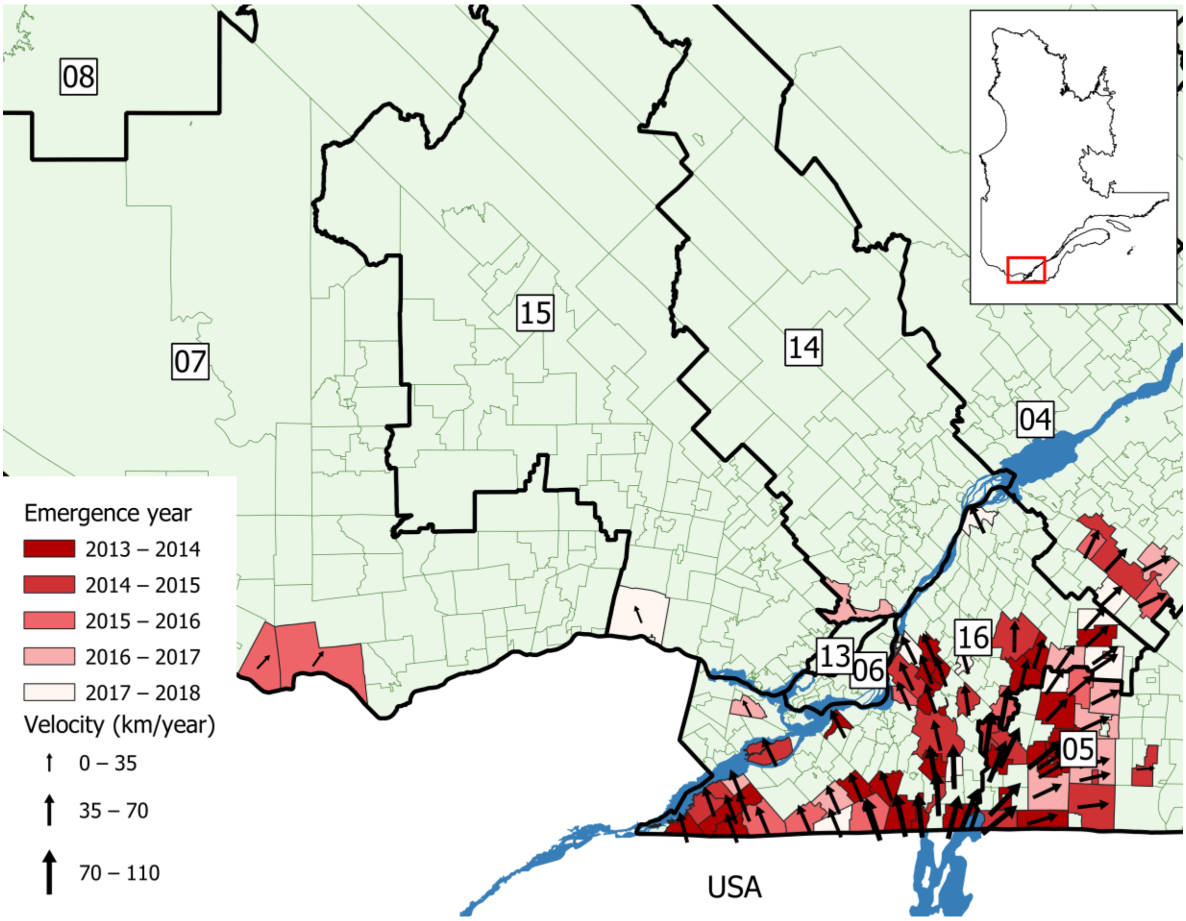

Figure S4. Lyme disease emergence velocity in Québec in municipalities having declared at least two cases in the last five years (n = 73) by date of notification (2013–2018). Sociosanitary regions: 04) Mauricie et Centre-du-Québec; 05) Estrie; 06) Montréal; 07) Outaouais; 08) Abitibi-Témiscamingue; 13) Laval; 14) Lanaudière; 15) Laurentides; 16) Montérégie.

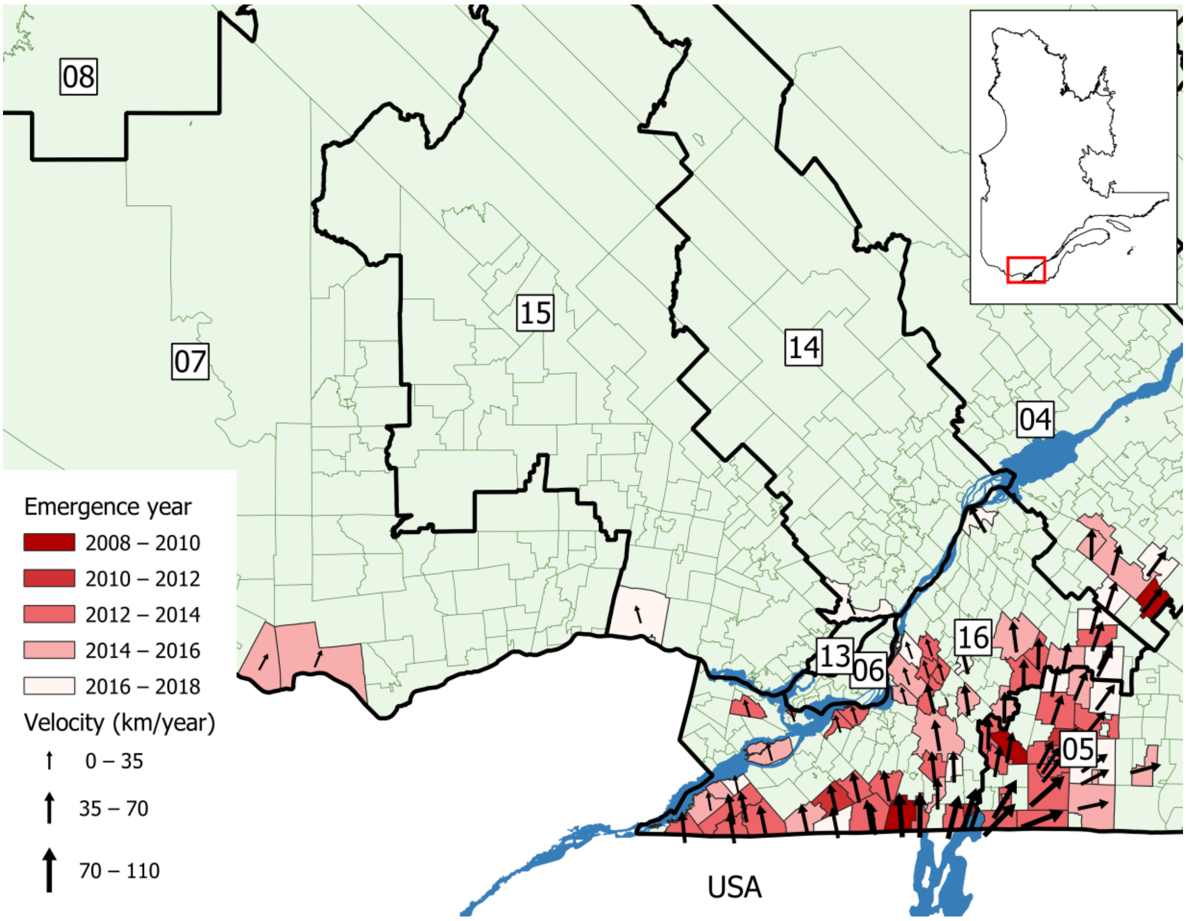

Figure S5. Lyme disease emergence velocity in Québec in municipalities having declared at least two cases over the whole study period (n = 75) by date of notification (2006–2018). Sociosanitary regions: 04) Mauricie et Centre-du-Québec; 05) Estrie; 06) Montréal; 07) Outaouais; 08) Abitibi-Témiscamingue; 13) Laval; 14) Lanaudière; 15) Laurentides; 16) Montérégie.

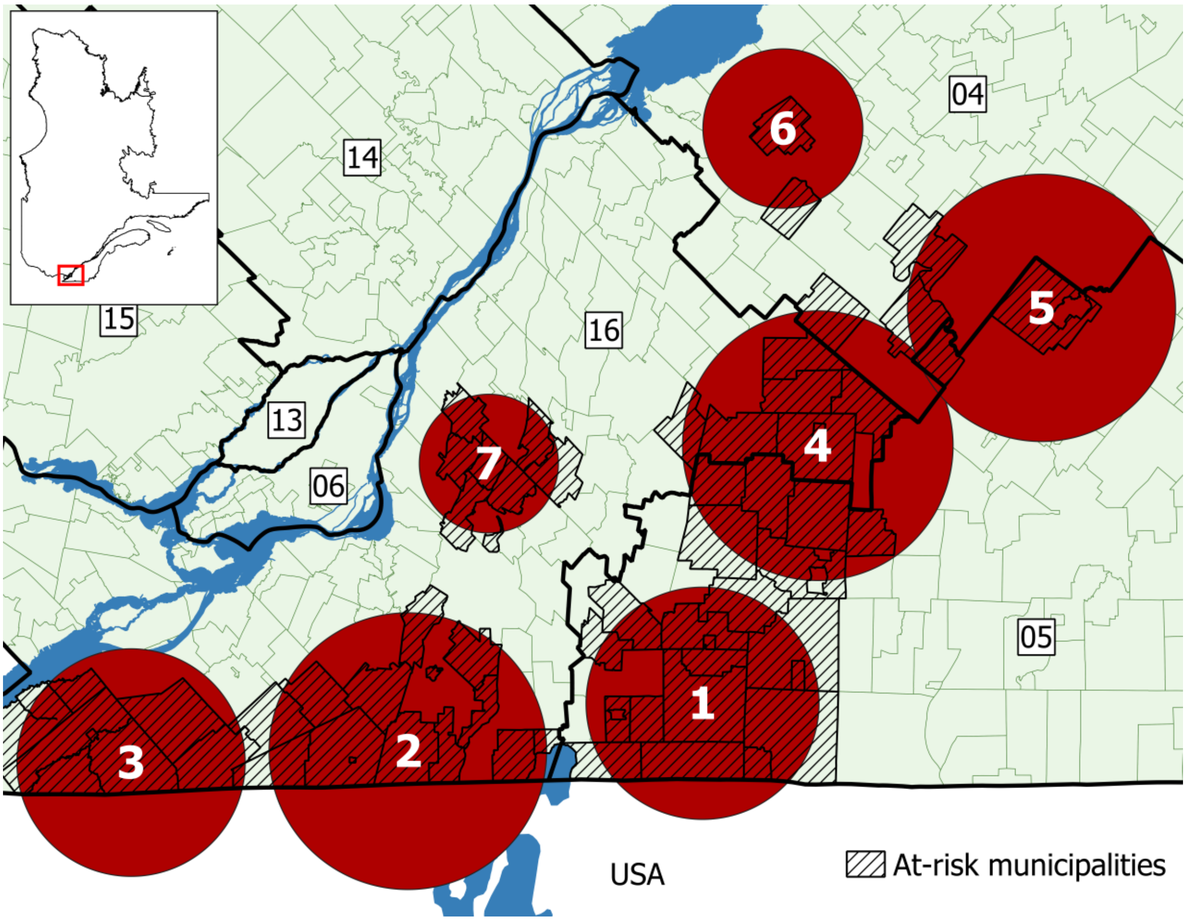

Figure S6. Spatiotemporal clusters of Lyme disease cases in Québec between 2006 and 2018 by date of symptom onset (*n* = 524 cases). Clusters are shown in order of appearance. If clusters appeared in the same time period, they were numbered by decreasing count of at-risk municipalities. Each circle represents a cluster no larger than 50 km in diameter, capturing no more than 15% of the population at-risk (underlying population). Sociosanitary regions: 04) Mauricie et Centre-du-Québec; 05) Estrie; 06) Montréal; 13) Laval; 14) Lanaudière; 15) Laurentides; 16) Montérégie.

Table S1. Spatiotemporal cluster variables of Lyme disease cases in Québec between 2006 and 2018 by date of symptom onset (n = 524 cases).

	**Clusters**						
	**1**	**2**	**3**	**4**	**5**	**6**	**7**
Radius (km)	20.80	24.81	20.41	23.97	23.67	14.05	12.33
Time period	2013-2018	2013-2018	2013-2018	2015-2018	2015-2018	2015-2018	2016-2018
Municipalities at-risk	15	14	8	14	4	2	7
Population at-risk	52 676	26 424	15 644	100 670	7 239	2 074	109 440
Declared cases	269	73	40	85	6	5	46
Expected cases	4.89	2.45	1.45	6.23	0.45	0.13	5.08
Annual cases/100 000	85.1	46.0	42.6	21.1	20.7	60.3	14.0
Relative risk	89.27	33.13	29.17	15.41	13.50	49.22	9.62
p-value	<0.001	<0.001	<0.01	<0.001	0.032	0.0016	<0.01

RR represents the relative risk of LD within the clusters relative to the risk outside the clusters.

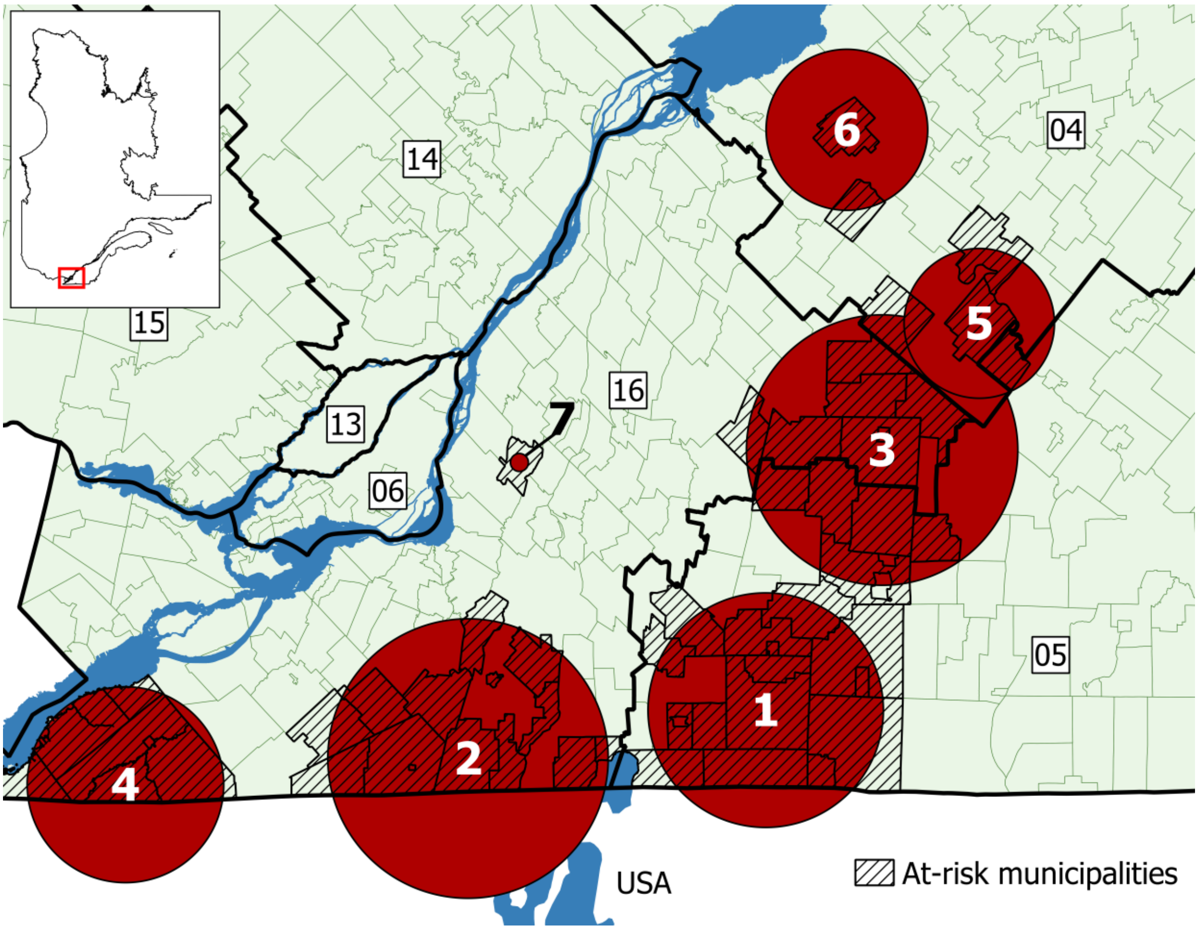

Figure S7. Spatial clusters of Lyme disease cases in Québec between 2006 and 2018 by date of symptom onset (*n* = 490 cases). Clusters are shown in order of number of municipalities at risk. Each circle represents a cluster no larger than 50 km in diameter, capturing no more than 15% of the population at-risk (underlying population). Sociosanitary regions: 04) Mauricie et Centre-du-Québec; 05) Estrie; 06) Montréal; 13) Laval; 14) Lanaudière; 15) Laurentides; 16) Montérégie.

Table S2. Spatial cluster variables of Lyme disease cases in Québec between 2006 and 2018 by date of symptom onset (n = 490 cases).

	**Clusters**						
	**1**	**2**	**3**	**4**	**5**	**6**	**7**
Radius (km)	20.80	24.81	23.80	17.40	13.14	14.05	0.00
Municipalities at-risk	15	14	13	7	3	2	1
Population at-risk	52 676	26 434	34 222	10 738	3 420	2 074	26 453
Declared cases	277	79	60	37	6	5	31
Expected cases	10.60	5.32	6.89	2.16	0.69	0.42	5.32
Annual cases/100 000	40.5	23.0	13.5	26.5	13.5	18.5	9.0
Relative risk	42.88	16.64	9.45	18.4	8.79	12.06	6.05
p value	<0.001	<0.001	<0.001	<0.001	0.011	0.0095	<0.001

RR represents the relative risk of LD within the clusters relative to the risk outside the clusters.

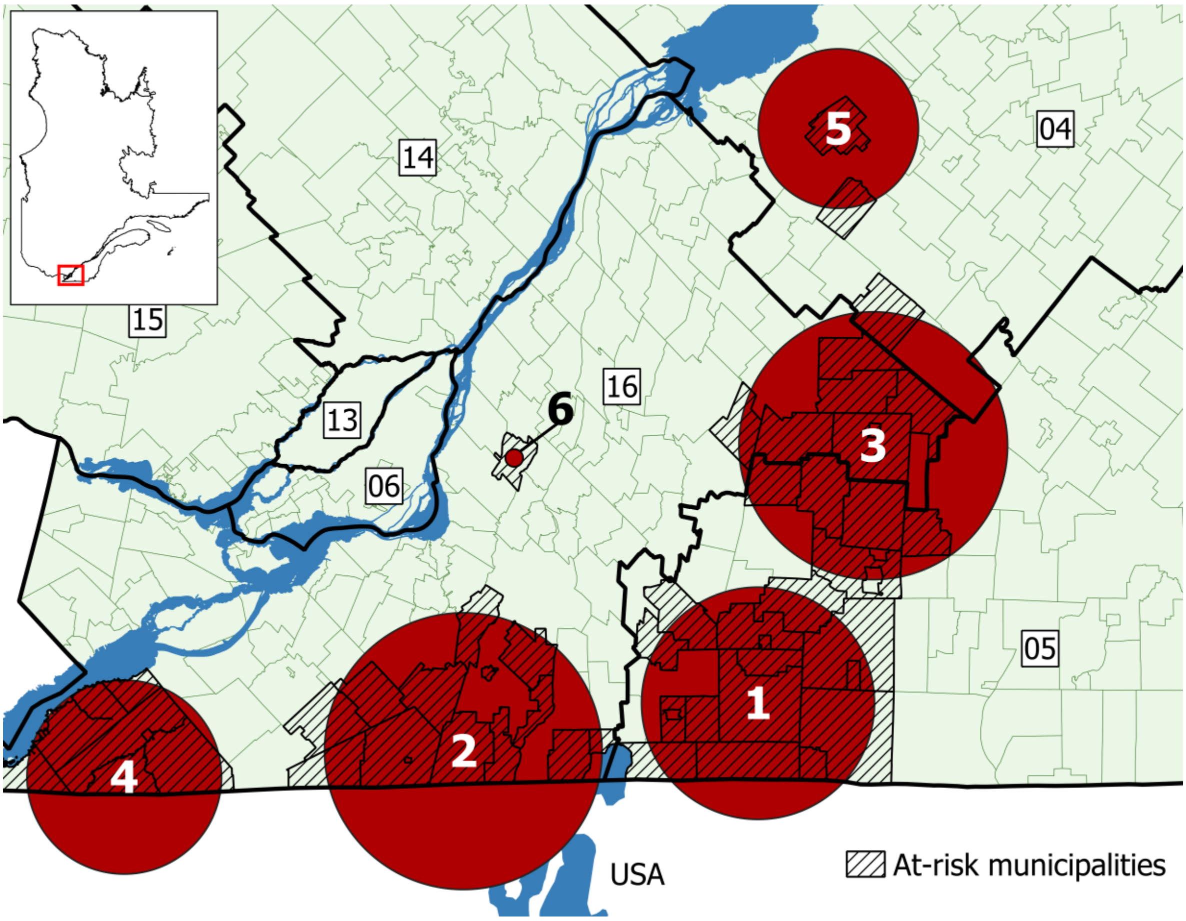

Figure S8. Spatial clusters of Lyme disease cases in Québec between 2006 and 2018 by date of notification (n = 506). Clusters are shown in order of number of municipalities at risk. Each circle represents a cluster no larger than 50 km in diameter, capturing no more than 15% of the population at-risk (underlying population). Sociosanitary regions: 04) Mauricie et Centre-du-Québec; 05) Estrie; 06) Montréal; 13) Laval; 14) Lanaudière; 15) Laurentides; 16) Montérégie.

Table S3. Spatial cluster variables of Lyme disease cases in Québec between 2006 and 2018 by date of notification (n = 506 cases).

	Clusters					
	**1**	**2**	**3**	**4**	**5**	**6**
Radius (km)	20.80	24.81	23.80	17.40	14.05	0.00
Municipalities at-risk	15	14	13	7	2	1
Population at-risk	52 675	26 423	34 222	10 739	2 074	26 453
Declared cases	290	81	62	37	5	31
Expected cases	10.14	5.09	6.59	2.07	0.40	5.09
Annual cases/100 000	42.4	23.6	13.9	26.5	18.5	9.0
Relative risk	46.90	17.79	10.19	18.80	12.60	6.31
p value	<0.001	<0.001	<0.001	<0.001	0.011	<0.001

RR represents the relative risk of LD within the clusters relative to the risk outside the clusters.
